# lncRNA SNHG1 induced by SP1 regulates bone remodeling and angiogenesis via sponging miR-181c-5p and modulating SFRP1/Wnt signaling pathway

**DOI:** 10.1186/s10020-021-00392-2

**Published:** 2021-11-03

**Authors:** Xiao Yu, Peng-Ze Rong, Meng-Sheng Song, Ze-Wen Shi, Gong Feng, Xian-Jun Chen, Lin Shi, Cheng-Hao Wang, Qing-Jiang Pang

**Affiliations:** 1 Department of Orthopedics, HwaMei Hospital, University of Chinese Academy of Sciences, Ningbo, 315000 Zhejiang Province China; 2 Ningbo Institute of Life and Health Industry, University of Chinese Academy of Sciences, No. 41 Xibei Street, Ningbo, 315000 Zhejiang Province China; 3 Ningbo University School of Medicine, Ningbo, 315211 Zhejiang Province China

**Keywords:** LncRNA SNHG1, miR-181c-5p, SFRP1, Wnt signal, Bone remodeling, Angiogenesis

## Abstract

**Background:**

We aimed to investigate the functions and underlying mechanism of lncRNA SNHG1 in bone differentiation and angiogenesis in the development of osteoporosis.

**Methods:**

The differential gene or proteins expressions were measured by qPCR or western blot assays, respectively. The targeted relationships among molecular were confirmed through luciferase reporter, RIP and ChIP assays, respectively. Alkaline phosphatase (ALP), alizarin red S (ARS) and TRAP staining were performed to measure the osteoblast/osteoclast differentiation of BMSCs. The viability, migration and angiogenesis in BM-EPCs were validated by CCK-8, clone formation, transwell and tube formation assays, respectively. Western blot and immunofluorescence detected the cytosolic/nuclear localization of β-catenin. Ovariectomized (OVX) mice were established to confirm the findings in vitro.

**Results:**

SNHG1 was enhanced and miR-181c-5p was decreased in serum and femoral tissue from OVX mice. SNHG1 directly inhibited miR-181c-5p to activate Wnt3a/β-catenin signaling by upregulating SFRP1. In addition, knockdown of SNHG1 promoted the osteogenic differentiation of BMSCs by increasing miR-181c-5p. In contrast, SNHG1 overexpression advanced the osteoclast differentiation of BMSCs and inhibited the angiogenesis of BM-EPCs, whereas these effects were all reversed by miR-181c-5p overexpression. In vivo experiments indicated that SNHG1 silencing alleviated osteoporosis through stimulating osteoblastogenesis and inhibiting osteoclastogenesis by modulating miR-181c-5p. Importantly, SNHG1 could be induced by SP1 in BMSCs.

**Conclusions:**

Collectively, SP1-induced SNHG1 modulated SFRP1/Wnt/β-catenin signaling pathway via sponging miR-181c-5p, thereby inhibiting osteoblast differentiation and angiogenesis while promoting osteoclast formation. Further, SNHG1 silence might provide a potential treatment for osteoporosis.

**Graphic abstract:**

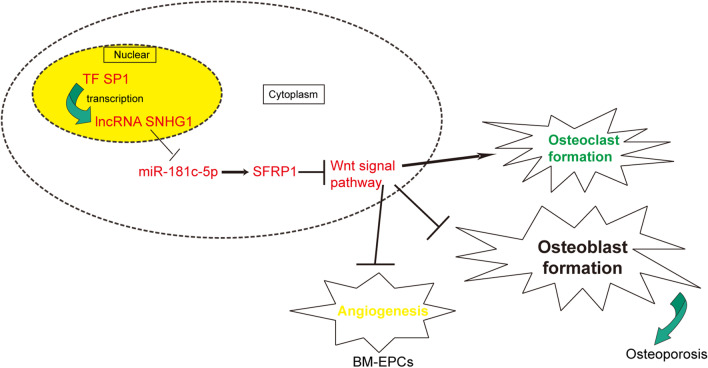

## Background

Osteoporosis (OP) is a bone disorder with the reduced bone mass, the degeneration of bone micro-architecture, bone weakening and an increasing risk of fracture (Anonymous 2000). The incidence of osteoporosis has been increasing dramatically recent years, which has become a public health problem in the worldwide (Cummings and Melton 2002). OP is associated with harmed bone formation and/or serious bone resorption (Montalcini et al. 2013). Besides, bone is a highly vascularized form of connective tissue and angiogenesis is required for bone formation (Kanczler and Oreffo 2008). It have proposed the concept of bone-vascular coupling, explaining that the angiogenesis of bone tissue is closely related to bone formation, bone remodeling and bone repair (Guo et al. 2021; Ma et al. 2021). The current treatments for osteoporosis are limited and usually combined with complications. Thus, it is urgent to explore the pathological mechanism of osteoporosis and find new effective treatment strategies.

Bone marrow mesenchymal stem cells (BMSCs) could differentiate into osteoblast, chondrocytes and adipocytes, which paly vital role in maintaining the balance between bone resorption and formation (Paspaliaris and Kolios 2019). It has been reported that changes in cell proliferation and differentiation capacity of BMSCs are also one key cause for osteoporosis (Liu et al. 2018; Qadir et al. 2020). Therefore, it is necessary to clarify the specific regulatory mechanism underlying the differentiation imbalance of BMSCs.

Long non-coding RNAs (lncRNAs) are a type of RNAs with the length exceeding 200 nucleotides. Many research suggested that lncRNAs are involved in the progression of a lot of diseases, including osteoporosis (Duan et al. 2020; Gao et al. 2020). Jiang et al. showed that lncRNA small nucleolar RNA host gene 1 (SNHG1) was up-regulated in the mice of osteoporosis, and its down-regulation promoted osteogenic differentiation of BMSCs by activating p38 MAPK signaling (Jiang et al. 2019). In previous studies, SNHG1 has been confirmed to be related to angiogenesis, and up-regulation of SNHG1 could promote the increase of angiogenesis in brain microvascular endothelial cells and glioma cells (Mi et al. 2020; Wang et al. 2018), implying SNHG1 was might be a modulator of angiogenesis. However, the roles and mechanism of SNGH1 in the osteoblast or osteoclast differentiation and angiogenesis need to be further explored.

MicroRNAs (miRNAs) are a type of no-coding RNAs (~ 22 nucleotides) which paly vital roles in the regulating of gene expression. Recently, miR-181c-5p has been reported to regulate bone metabolism and differentiation in osteoporosis development. For example, miR-181c-5p was downregulated in the bone tissue of OVX mice, and overexpression of miR-181c-5p promoted the differentiation and mineralization of osteoblasts (Ma et al. 2020). Murodumi et al. revealed that the increase of miR-181c-5p could induce osteogenic differentiation and calcification in human jawhone-derived osteoblastic cells by targeting Nothc2 (Murodumi et al. 2020). Whereas, whether there is correlation between miR-181c-5p and SNHG1 in OP remains to be studied.

Frizzled-related protein-1 (SFRP1), one of the most important proteins in the SFRPs family, plays vital roles in osteoblast differentiation, trabecular bone formation and bone fracture healing (Bodine et al. 2009, 2004). It was reported that SFRP1 was involved in OP through negatively regulating the survival of human osteoblast and osteocyte survival (Amjadi-Moheb et al. 2018; Zhang et al. 2018). Wnt signaling pathway serves a vital role in regulating osteoblastic differentiation, skeletal development and bone homeostasis (Duan and Bonewald 2016). SFRP1 could inhibit the Wnt signaling pathway by competitively binding to the Frizzled receptor (Hausler et al. 2004). However, whether SFPR1 is involved in the process by which SNHG1/miR-181c-5p regulates OP remains to be elucidated.

LncRNAs are transcribed from DNA sequences in accordance with the rules of base complementary pairing and are regulated by multiple transcription factors. During bioinfomatics analysis, we have found the recognition sequences of SP1 on the promoter region of SNHG1. Importantly, we also found the putative targeting site of miR-181c-5p on SNHG1 and SFRP1. In the present study, we explored the functions and mechanisms among SP1, SNHG1, miR-181c-5p and SFRP1 in bone differentiation and angiogenesis against the background of the above studies. Finally, our data demonstrated that SP1-induced SNHG1 regulates osteoblast differentiation, osteoclast and angiogenesis via SFRP1-dependent Wnt signaling pathway by interacting miR-181c-5p.

## Methods

### Animal

Forty-eight healthy 8-week-old C57BL/J6 female mice were obtained from SLAC laboratory Animal Co., Ltd. (Shanghai, China). Mice were divided into four groups: sham, surgical ovariectomy (OVX), OVX + sh-SNHG1 and OVX + sh-SNHG1 + miR-181c-5p inhibitor groups and maintained in pathogen-free facilities. After 1 week of acclimatization, bilateral OVX and sham operations were performed as previously reported (Jiang et al. 2019). Eight weeks after the operation, mice were sacrificed and mouse femurs and blood samples were harvested and stored for further detection. In other groups, after successful OVX modeling, mice were injected with lentiviral vector of sh-SNHG1 (5 μL) or miR-181c-5p inhibitor (5 μL) by tail vein once a week for four weeks. All protocols were approved by the Animal Care Committee of the Hwa Mei Hospital, University of Chinese Academy of Sciences and conducted under the NIH guidelines for animal use.

### Cell culture and induction

BMSCs were isolated as previously described (Ubellacker et al. 2017; Zhao et al. 2020). In brief, BMSCs extracted from the femurs and tibiae of 9–10-week-old mice and cultured in Dulbecco’s modified Eagles medium (DMEM, Gibco, USA) supplemented with 10% fetal bovine serum (FBS), and 1% penicillin–streptomycin (Grand Island, NY, USA). The isolation of mouse bone marrow-derived endothelial progenitor cells (BM-EPCs) was performed as previously describe, and cultured in Endothelial Cell Growth Medium2 (EGM-2, Lonza, Anaheim, CA) supplemented with 5% mouse epidermal growth factor (EGF), vascular endothelial growth factor (VEGF), FGF-B, ascorbic acid, and heparin (Lonza). Cells were cultured at 37 °C in a humidified condition of 95% O_2,_ and the medium was changed every 2 days.

To induce osteogenic differentiation, BMSCs were seeded at a density of 1 × 10^5^/well in six-well plates and cultured in osteogenic induction medium (DMEM supplemented with 10 mM β-glycerophosphate, 100 μM l-ascorbic acid 2-phosphate, 2 mM l-glutamine, 100 nM dexamethasone and 10% FBS for 3 weeks. For osteoclast differentiation, BMSCs were seeded at a density of 1 × 10^5^/well in six-well plates and cultured with DMEM containing 50 ng/mL (macrophage-colony stimulating factor) M-CSF (416-ML, R&D Systems) for 3 days. Then the cells were cultured with DMEM with M-CSF (50 ng/mL), RANKL (50 ng/mL, 315-11, Peprotech) for 10 days. The culture medium was changed every 3 days.

### Cell transfection

The short hairpin RNA (shRNA) targeting SNHG1 and SP1 were designed by GenePharma (Shanghai, China). The miR-181c-5p mimics, inhibitors and corresponding negative control were purchased from GenePharma (Shanghai, China). To construct the SNHG1 and SFRP1 overexpressing vectors, the full-length sequences of SNHG1 and SFRP1 were amplified and inserted into pcDNA3.1 vector (Invitrogen). BMSCs or BM-EPCs (1 × 10^4^ cells/well) were seeded in 24-well plates to grow 70% confluent, then transfection was conducted with RNAs (50 nM) or vectors (1 µg per well) with Lipofectamine 3000 (Invitrogen) according to the protocol.

### Quantitative real-time PCR (qPCR)

Total RNA was isolated using Total RNA kit (Tiangen, Beijing, China). The first-strand cDNA was synthesized through a miScript Reverse Transcription kit (Thermo Fisher Scientific Co., Ltd. Shanghai, China). Then, qPCR was conducted on a Real-Time PCR system (7500; Thermo) with miScript SYBR® Green PCR kit (Qiagen, Inc.). The amplification conditions were 95 °C for 5 min, followed by 40 cycles of 30 s of 94 °C and 30 s of 60 °C, 72 °C for 10 min. Relative levels of RNAs were quantified by the 2^−ΔΔCq^ method and normalized to those of GADPH. The primer sequences used in this experiment were showed in Table 1.

**Table 1:** Primers used for qPCR assay

Gene	Sequence (5′ → 3′)
SNHG1	Forward: TCCTTGTTCGGGGTTTGAGG
	Reverse: ACAGCACCCTGACTACAAGC
miR-181c-5p	Forward: GCCGAGAACATTCAACCTGTCG
	Reverse: GTCGTATCCAGTGCAGGGTCCGAGGTATTCGCACTG GATACGACACTCAC
ALP	Forward: AACCCAGACACAAGCATTCC
	Reverse: CCAGCAAGAAGAAGCCTTTG
OCN	Forward: AATCCAACGGTGTGAAGAGC
	Reverse: CCTGCGTGGAGTATCCATCT
RUNX2	Forward: AGATGGGACTGTGGTTACCG
	Reverse: GGACCGTCCACTGTCACTTT
NFATc1	Forward: GGTGCTGTCTGGCCATAACT
	Reverse: GCGGAAAGGTGGTATCTCAA
RANKL	Forward: CCTCACCATCAATGCTGCCA
	Reverse: TGTAGGTACGCTTCCCGATG
CTSK	Forward: CTTCCAATACGTGCAGCAGA
	Reverse: TTGCATCGATGGACACAGAG
VEGF	Forward: GAGAGAGGCCGAAGTCCTTT
	Reverse: TTGGAACCGGCATCTTTATC
SP1	Forward: TGGGTACTTCAGGGATCCAG
	Reverse: TGAGGCTCTTCCCTCACTGT
SFRP1	Forward: AAGCGAGTTTGCACTGAGGA
	Reverse: TACTGGCTCTTCACCTTGCG
Wnt3a	Forward: GGGCACTAACAAGTCGGGT
	Reverse: GGGCATGATCTCCACGTAGT
β-catenin	Forward: ACAGGGTGCTATTCCACGAC
	Reverse: AGCAACTGCACAAACAATGG
U6	Forward: CTCGCTTCGGCAGCACA
	Reverse: AACGCTTCACGAATTTGCGT
GAPDH	Forward: AGCCCAAGATGCCCTTCAGT
	Reverse: CCGTGTTCCTACCCCCAATG

### Western blot

Total proteins were isolated with RIPA lysis buffer (Beyotime, China) supplemented with proteinase inhibitors and concentration was detected using the BCA kit (#23225; Pierce, Inc.). 20 µg total proteins were electrophoresed by 10% SDS-PAGE and transferred onto PVDF membrane (Millipore, Burlington, MA, USA). After blocking (5% milk, 37 °C, 2 h), membranes were incubated with respective primary antibodies (Abcam, Cambridge, UK) against RUNX2 (ab192256, 1:2000), OCN (ab93876, 1:500), ALP (ab224335, 1:2000), NFATc1 (ab25916, 1:1000,), RANKL (ab239607, 1:500), CTSK (ab207086, 1:500), VEGF (ab69479, 1:200), SFRP1 (ab126613, 1:1000), Wnt3a (ab219412, 1:500), β-catenin (ab16051, 1:4000), β-actin (ab6276,1: 5000) and β-tubulin (ab6046, 1:500) at 4 °C overnight. Subsequently, the membrane was incubated with appropriate secondary HRP antibody and exposed using an ECL kit (EMD Millipore), and band intensity was quantified by ImageJ software.

### Alkaline phosphatase (ALP) staining

After osteogenic differentiation, BMSCs were collected and washed using phosphate-buffered saline (PBS). After fixed by 4% paraformaldehyde (PFA), BMSCs were stained using an ALP staining kit (CoWin Biotech, Beijing, China). Images were captured using a microscope (Nikon Corporation).

### Alizarin red S (ARS) staining

BMSCs were placed on glass slid and fixed with 4% PFA (RT, 20 min), and washed using distilled water. After that, cells were incubated in 2% Alizarin red staining reagent (sigma) for 15 min. Subsequently, after washing, images were captured using a microscope (Nikon Corporation).

### TRAP staining

BMSCs were fixed by 4% PFA (15 min) and washed using PBS (3 times, 20 min). Then, the TRAP staining fluid was added and cells were incubated in TRAP staining fluid (Sigma) at RT for 1 h. After washing, images were captured using an inverted microscope (Nikon). TRAP-positive BMSCs with more than three nuclei were classified as osteoclasts.

### Transwell assay

After treatment, BMSCs or BM-EPCs (4 × 10^4^) were planted to the upper transwell chamber (8 mm, BD Biosciences) coated with BD BioCoat Matrigel. The lower chambers were coated with medium (600 μL) containing 10% FBS. After incubation for 12 h, the migrated cells were fixed uisng 20% methanol and stained by violet crystalline. Representative images were captured through the optical microscope (Nikon).

### Cell adhesion assay

The 96-well plate was coated uisng 5 μg/mL fibronectin (Millipore, CA) and 2% bovine serum albumin (to avoid non-specific cell binding) for 2 h at 37 °C. BMSCs (5 × 10^4^ cells/well) were seeded for 1 h. After that, non-adherent BMSCs were removed. Adherent BMSCs were fixed by 4% PFA and staining using 1% crystal violet. Representative images were obtained using a microscope, and cell number was counted.

### Tube formation assay

Cold Matrigel (Matrigel, BD biosciences, CA, USA) was coated into 96-well plate and incubated at RT for 40 min. Then, BM-EPCs (2 × 10^4^ cells/well) were added. Six hours later, representative images in each well were randomly photographed using a Nikon inverted microscope. The tube length was quantified by Image Pro Plus 6.0 software.

### Colony formation assay

Cell (2 × 10^6 ^cells/well) were planted into 6-well plates in triplicate and cultured at 37 °C and 5% CO_2_ in a humidified chamber for 2 weeks. The cell culture was terminated and the culture medium was discarded. Then, cells were fixed using 4% paraformaldehyde and stained with 1% crystal violet staining solution for 10 min. The colony was observed with an optical microscope.

### Cell counting kit (CCK)-8 assay

Cells (2 × 10^3^ cells/well) were seeded into 96-well plates and cultured at 37 °C. 10 μL of CCK-8 solution (Dojindo Co. Tokyo, Japan) was added the wells after 0, 6, 12, 24 and 48 h, respectively. Then cells were incubated for 3 h at 5% CO_2_. The optical density (OD) at 450 nm were detected by a microplate reader (Bio-Rad, California, USA). A curve was plotted based on the OD value.

### RNA binding protein immunoprecipitation (RIP) assay

BMSCs were transfected with miR-181c-5p mimics or NC for 48 h. Cells were harvested and RIP assay was conducted through an EZ-Magna RNA Immunoprecipitation Kit (Millipore Corporation, USA) following manufacturer’s instructions. Antibodies against Ago2 (ab186733, Abcam) and IgG (ab172730, Abcam) were purchased from Abcam. Finally, the binging products were quantified by qPCR.

### Chromatin immunoprecipitation (ChIP) assay

ChIP was conducted with an EZ ChIP™ Chromatin Immunoprecipitation Kit (Millipore). Briefly, the cross-linked chromatin DNA was ultrasonic vibrated io 200–500 bp fragments and immunoprecipitated with anti-SP1 (ab231778, Abcam) and anti-IgG (ab172730, Abcam) antibodies. Lastly, the immunoprecipitated DNA was quantified by qPCR. And results were normalized relative to the input IgG.

### Immunofluorescence (IF)

BMSCs were cultured on 24-well plates at 37 °C for 1 week, β-catenin was detected by IF. The cells were fixed with 4% PFA for 30 min at RT, rinsed 3 times with PBS and blocked with 1% bovine serum albumin for 30 min at room temperature. Then, the cells were incubated overnight at 4 °C with primary antibody against β-catenin (ab32572, 1:30, Abcam). Subsequently, the cells were incubated with fluorescence-conjugated secondary antibody for 2 h at room temperature. Nuclei were stained with DAPI (OriGene Technologies, Inc.). After rinsing with PBS, images were randomly photographed using a fluorescence microscope (Olympus Corporation).

### Luciferase reporter assay

The putative binding sites for SP1 containing SNHG1 promoter regions F2 and the potential miR-181c-5p binding sites or wild/mutant (WT/MUT) of SNHG1/SFRP1 were inserted into pGL3 plasmid (Promega, USA), and named as pGL3F2, SNHG1-WT, SNHG1-MUT, SFRP1-WT, SFRP1-MUT. Cells were co-transfected with pGL3F2 along with SP1 vector or an empty vector, SNHG1-WT/MUT or SFRP1-WT/MUT along with miR-181c-5p mimics or mimics NC, for 2 days using Lipofectamine 3000. The luciferase activities were detected using Dual Luciferase Assay System (Promega).

### Bone histomorphometric analysis

The mouse femurs were harvested and fixed in 4% paraformaldehyde for 2 days, and decalcified uisng 10% ethylenediaminetetraacetic acid, embedded in paraffin, then cross-sectioned (5 μm) on the rotary microtome (RM2235, Leica, Germany). Hematoxylin and eosin (HE) staining was conducted as the previous study (Zhang et al. 2018). The differentiation of osteoclasts was measured by TRAP staining. Bone sections were labeled with Masson staining according to the manufacturer’s protocol (Sigma-Aldrich, Missouri, USA). Immunohistochemical (IHC) staining was performed as previously described (Zhao et al. 2021). The primary antibody against NFATc1 (sc-7294, 1:200) was purchased from Santa Cruz Biotechnology (Santa Cruz, CA, USA). Five fields were randomly selected per section and photographed.

### Statistical analysis

All data was analyzed using SPSS 22.0 software (IBM, NY). Mean comparisons were evaluated by the Student’s t-test or one-way analysis of variance (ANOVA) with post hoc contrasts by Tukey test. Data were expressed with mean ± standard deviation. A *Ρ* < 0.05 was considered as statistically significant.

## Results

### The dysregulation of lncRNA SNHG1 and miR-181c-5p in osteoporotic mice

**Fig. 1:**
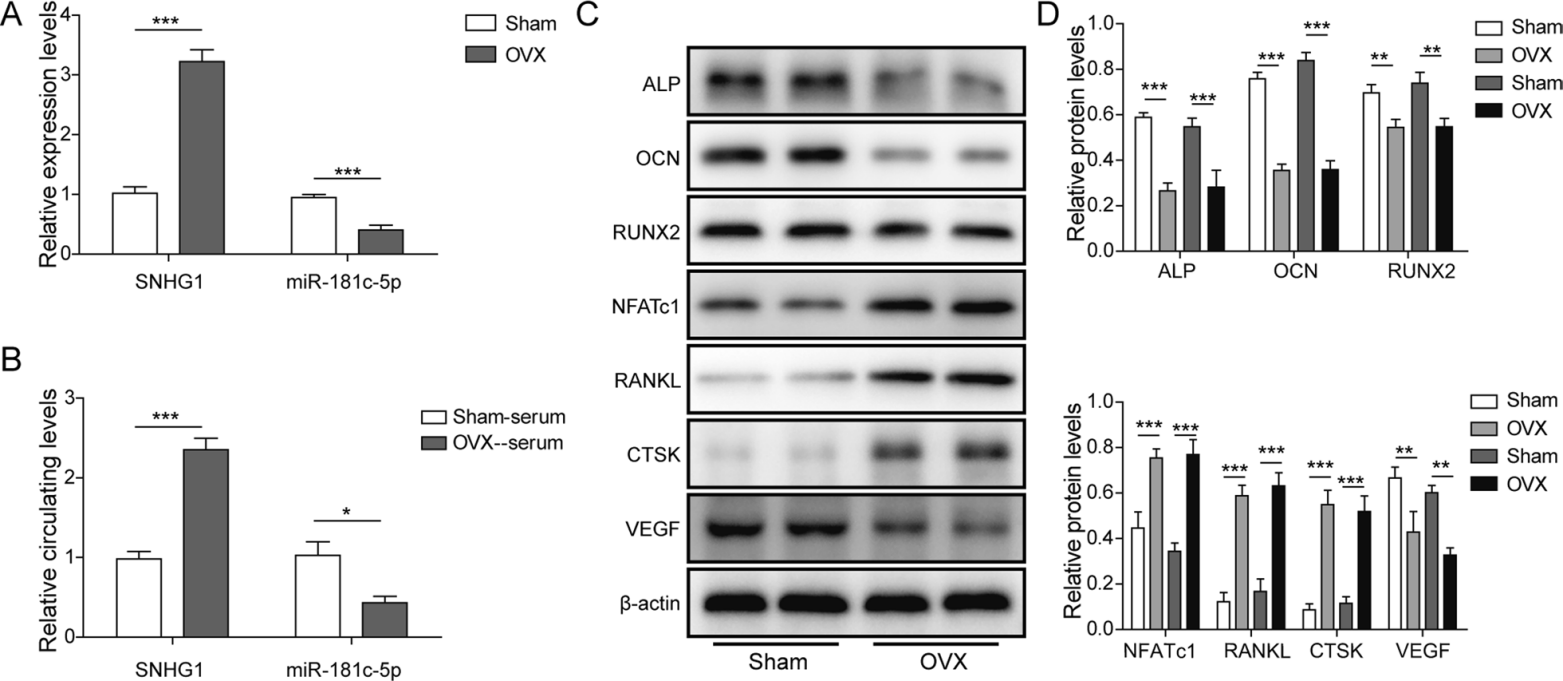
Dysfunctional expression of lncRNA SNHG1 and miR-181c-5p in OVX mice. Sixteen mice were randomly divided into sham group or ovariectomy (OVX) group. Eight weeks after the operation, mice were euthanized, femurs and serum were collected. **A** The SNHG1 and miR-181c-5p expressions in femoral tissue of were detected through qPCR. **B** The circulating levels of SNHG1 and miR-181c-5p in serum were measured through qPCR. **C** and **D** The protein levels of ALP, OCN, RUNX2, VEGF, NFATc1, RANKL and CTSK in femoral tissue were assessed by western blot assay. Per lane was loaded with protein collected from every four mice. **P < 0.01, ***P < 0.001 *vs*. sham mice

The postmenopausal osteoporotic mice model was established bilateral ovariectomy in female mice (OVX mice). After OVX surgery, SNHG1 and miR-181c-5p levels were evaluated in OVX mice and shame mice. The relative circulating level of SNHG1 in the serum and femoral tissue of OVX mice was significantly higher than those in the sham mice, while the expression of miR-181c-5p was obviously down-regulated (Fig. 1A and B). Moreover, the protein levels of ALP, OCN, RUNX2 and VEGF decreased significantly in femoral tissue of OVX group compared with sham group, while the levels of NFATc1, RANKL and CTSK increased significantly (Fig. 1C and B). These data suggested that SNHG1 and miR-181c-5p were dysregulated in osteoporosis.

### LncRNA SNHG1 could bind to miR-181c-5p to repress its expression

**Fig. 2:**
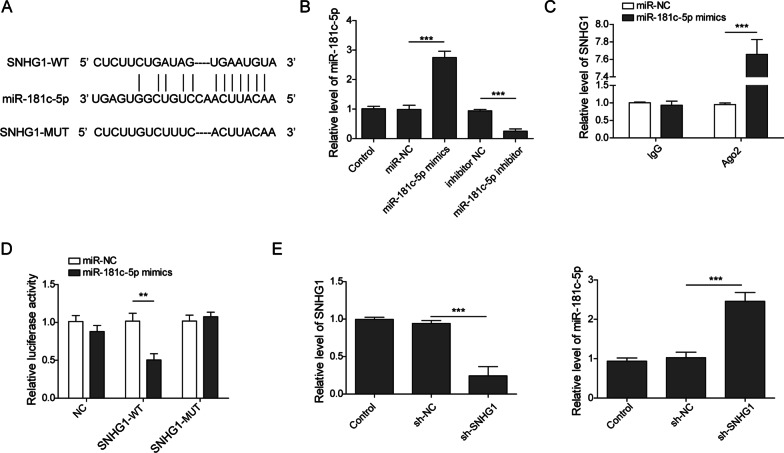
LncRNA SNHG1 served as a sponge for miR-181c-5p. **A** starBase analysis showed the potential binding sites for SNHG1 and miR-181c-5p. **B** The BMSCs were transfected with miR-181c-5p mimics, miR-181c-5p inhibitor or their negative controls for 48 h, then the level of miR-181c-5p was measured suing qPCR. **C** The RIP assay performed to determine the interaction between SNHG1 and miR-181c-5p in BMSCs. **D** Luc-SNHG1-WT or Luc-SNHG1-MUT plasmids were co-transfected with miR-181c-5p mimics or negative control for 24 h, the luciferase activities in each group were measured. **E** The BMSCs were transfected with sh-SNHG1 or sh-NC, then the relative levels of SNHG1 and miR-181c-5p were measured through qPCR. **P < 0.01, ***P < 0.001

Generally, lncRNAs were reported to function as the molecular sponge of miRNA. Here, we further investigated the downstream regulatory mechanism of SNHG1. As shown in Fig. 2A, starbase software (http://starbase.sysu.edu.cn/) predicted 7 bp continuous complementary bases between SNHG1 and the “seed region” of miR-181c-5p. In order to validate the potential molecular mechanism between of the two, miR-181c-5p was overexpressed by transfection miR-181c-5p mimics and downregulated by transfection miR-181c-5p inhibitor in BMSCs (Fig. 2B). RIP assay disclosed that SNHG1 level was enriched in RNA-complex pulled down by Ago2 in BMSCs transfected with miR-181c-5p mimics (Fig. 2C). In addition, the luciferase activity in SNHG1-WT group was inhibited by miR-181c-5p mimics, whereas there was no significant change in luciferase activity in MUT-SNHG1 group (Fig. 2D). Expectedly, qPCR data demonstrated that sh-SNHG1 transfection led to the reduction of SNHG1 and greatly elevated of miR-181c-5p in BMSCs (Fig. 2E). Therefore, these results implicated that SNHG1 play as a sponge of miR-181c-5p.

### Silencing of lncRNA SNHG1 promoted osteogenic differentiation by regulating miR-181c-5p

**Fig. 3:**
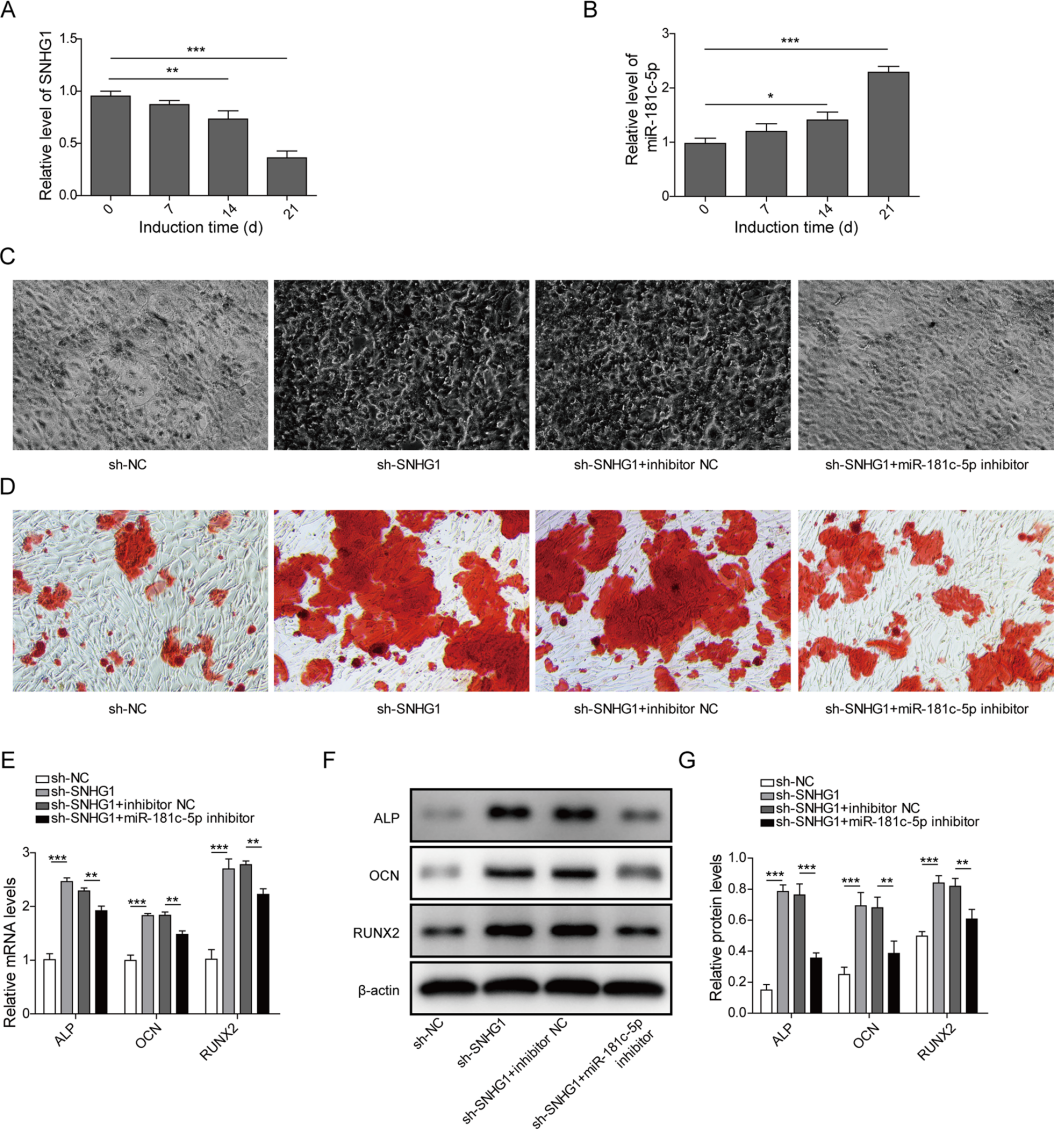
LncRNA SNHG1 knockdown promoted the osteogenic differentiation of BMSCs through regulation of miR-181c-5p. **A** and **B** The levels of lncRNA SNHG1 and miR-181c-5p in BMSCs were determined using qPCR at 0, 7, 14 and 21 days after osteogenic induction. BMSCs were transfected with sh-NC, sh-SNHG1, sh-SNHG1 with inhibitor NC, or sh-SNHG1 with miR-181c-5p inhibitor and induced for osteogenic differentiation. **C** and **D** ALP staining and ARS were performed at day 14 and 21 after osteogenic differentiation. **E–F** 21 days after osteogenic induction, the mRNA and protein levels of ALP, OCN and RUNX2 in BMSCs were detected by qPCR and western blot. *P < 0.05, **P < 0.01, ***P < 0.001

The levels of lncRNA SNHG1 and miR-181c-5p in BMSCs were detected by qPCR after the osteogenic induction for 0, 7, 14, and 21 days. As shown in Fig. 3A, lncRNA SNHG1 expression was gradually down-regulated in osteogenic induction, exhibiting a significant decrease at 7 days. However, miR-181c-5p expression showed the opposite tendency (Fig. 3B). To verify whether the biological effect of lncRNA SNHG1 on BMSCs osteogenic differentiation is dependent on the regulation of miR-181c-5p, we transiently knockdown miR-181c-5p in lncRNA-SNHG1 stably silencing BMSCs. After induction for 21 days, osteogenic differentiations of BMSCs were determined. ALP staining revealed that silencing of lncRNA SNHG1 increased ALP activity, while miR-181c-5p inhibitor could rescue this effect (Fig. 3C). In addition, the mineralized bone matrix formation, which was measured by ARS staining, was increased in the sh-SNHG1 group, whereas it was decreased after miR-181c-5p inhibitor transfection (Fig. 3D). Moreover, sh-SNHG1 increased the mRNA and protein levels of ALP, OCN and RUNX2, while these effects could be partially repaired by miR-181c-5p inhibitor (Fig. 3E–G). Collectively, these results strongly suggested that knockdown of lncRNA SNHG1 promoted osteogenic differentiation of BMSCs by increasing miR-181c-5p.

### Overexpression of lncRNA SNHG1 facilitated osteoclast formation of BMSCs by regulating miR-181c-5p

**Fig. 4:**
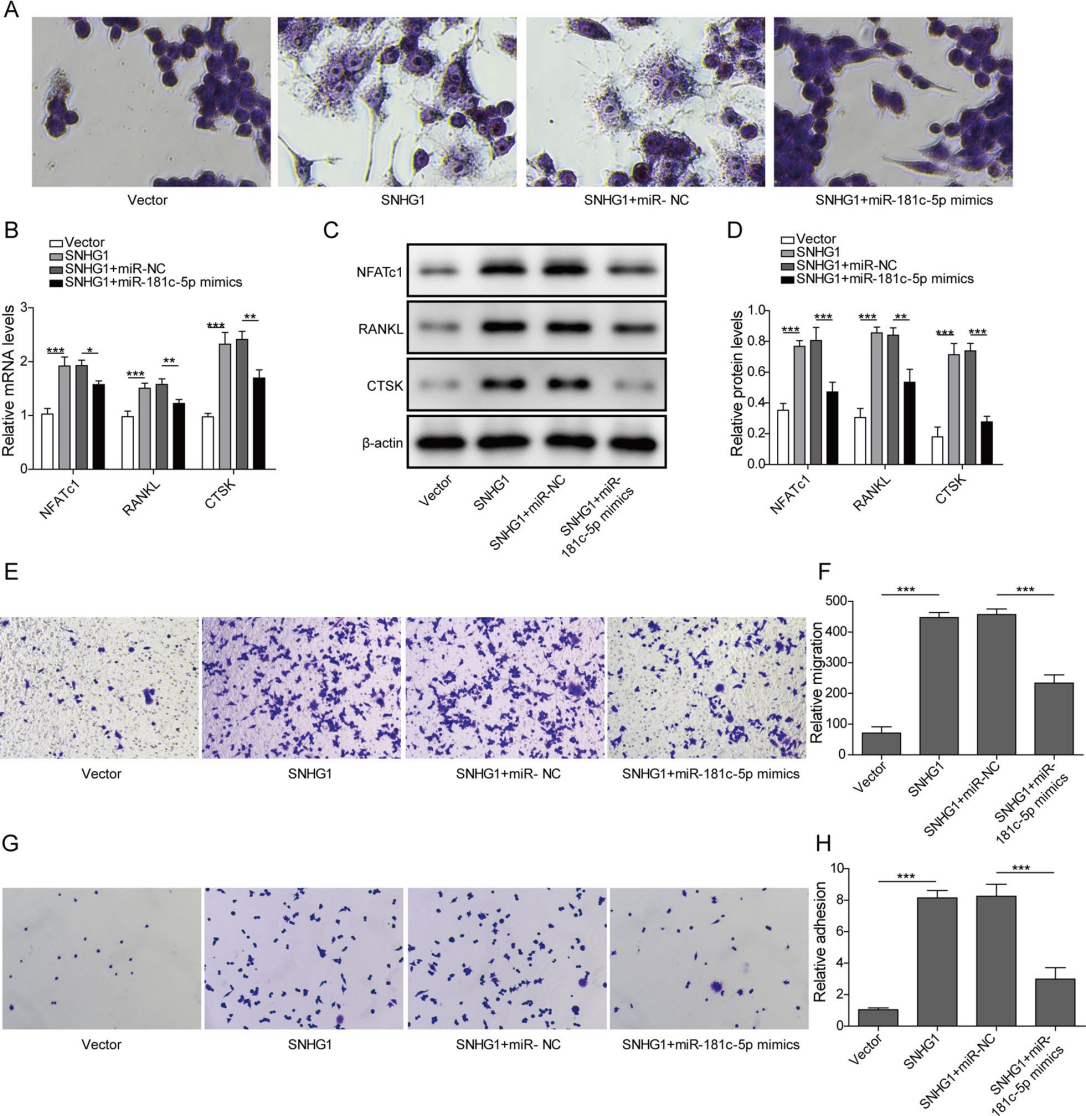
LncRNA SNHG1 overexpression promoted osteoclast differentiation by targeting miR-181c-5p. The BMSCs were transfected by vector-NC, SNHG1 vector, SNHG1 vector with miR-NC, or SNHG1 vector with miR-181c-5p mimics, and induced with M-CSF and RANKL for 10 days. **A** Representative images of TRAP staining in four groups. **B–D** After induction, the mRNA and protein expression of NFATc1, RANKL and CTSK in BMSCs were measured through qPCR and western blot. **E** and **F** Cell migration of treated BMSCs was determined using Transwell assay. **G–H** Adhered cells were stained with crystal violet and cell number was counted. *P < 0.05, **P < 0.01, ***P < 0.001

To assess whether lncRNA SNHG1 regulated the osteoclast formation via targeting miR-181c-5p, BMSCs were transfected with SNHG1 vector alone or co-transfected with SNHG1 vector and miR-181c-5p mimics. Then, the transfected cells were then exposed to medium containing M-CSF and RANKL to induce osteoclast differentiation. TRAP staining analysis showed that overexpression of miR-181c-5p attenuated the increased number of TRAP-positive multinucleated cells caused by SNHG1 vector (Fig. 4A). The mRNA and protein levels of osteoclast marker genes including NFATc1, RANKL and CTSK were up-regulated in response to lncRNA SNHG1 overexpression, while co-overexpressing of miR-181c-5p neutralized this promoting effects (Fig. 4B and D). Furthermore, upregulation of lncRNA SNHG1 aggravated the migration ability of BMSCs, while co-overexpression of miR-181c-5p greatly reversed this role (Fig. 4E and B). Similarly, miR-181c-5p mimics significantly restrained the promoting effect of SNHG1 overexpression on the adhesion ability of BMSCs (Fig. 4G and H). Overall, these data indicated that lncRNA SNHG1 induced osteoclast differentiation of BMSCs through miR-181c-5p.

### LncRNA SNHG1 suppressed the proliferation, migration and tube formation of BM-EPCs by inhibiting miR-181c-5p

**Fig. 5:**
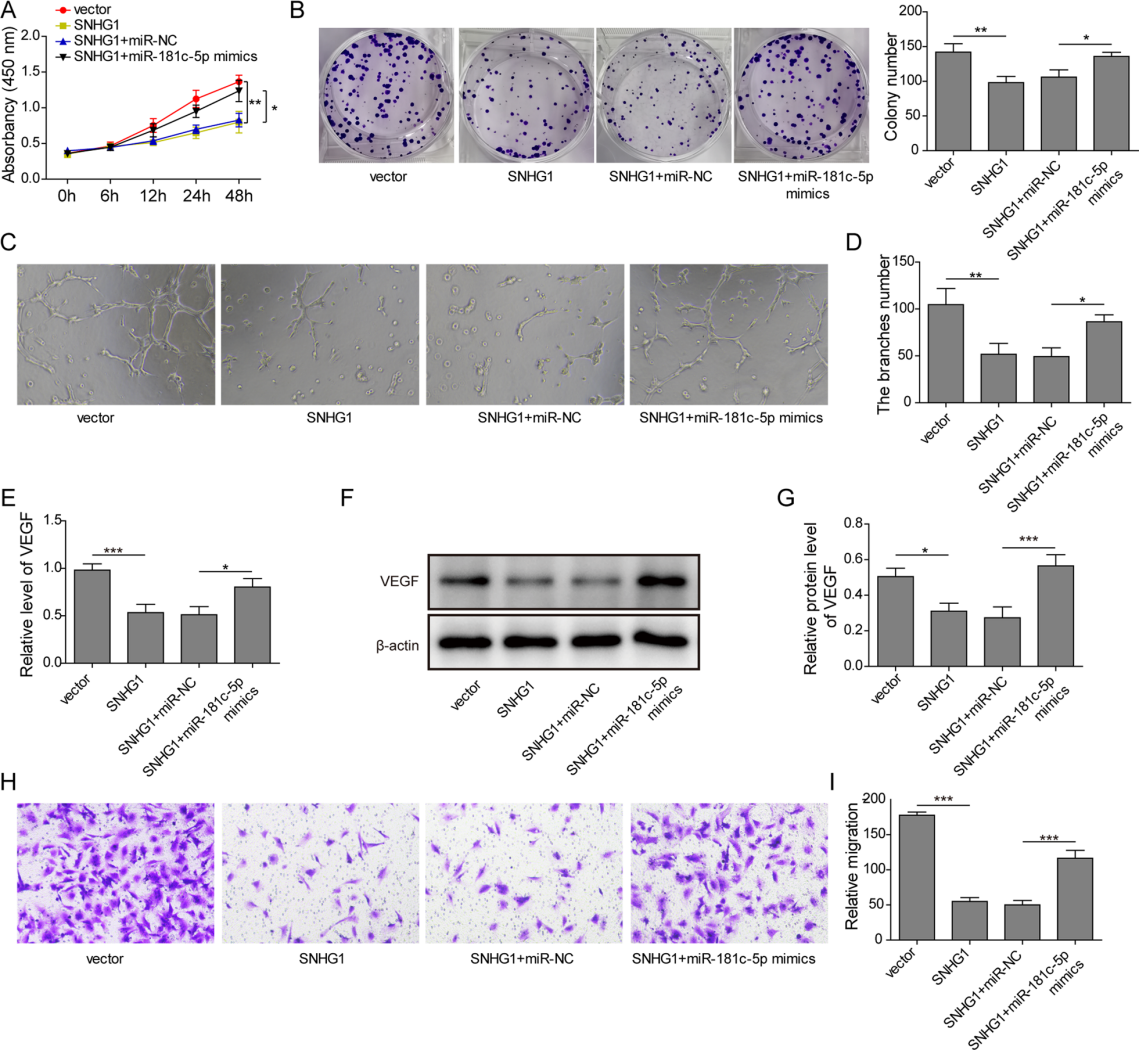
Overexpression of lncRNA SNHG1 inhibited tube formation of BM-EPCs via targeting miR-181c-5p. The BM-EPCs were transfected with vector-NC, SNHG1 vector, SNHG1 vector with miR-NC, or SNHG1 vector with miR-181c-5p mimics for 48 h. **A** and **B** The cell proliferation and colony were detected by CCK-8 assay and colony formation assay. **C** After cultured with Matrigel for 6 h, the images of tubular structures of transfected BM-EPCs. **D** The tube lengths in every group. **E** The mRNA expression level of VEGF in BM-EPCs was detected by qPCR. **D** and **E** The protein expression level of VEGF was measured through western blot. **F** and **G** Cell migration was detected using Transwell assay. *P < 0.05, **P < 0.01, ***P < 0.001

We then studied the function of SNHG1/miR-181c-5p axis in angiogenesis. The BM-EPCs were transfected with SNHG1 vector alone or co-transfected with SNHG1 vector and miR-181c-5p mimics. The CCK-8 and clone formation assays showed greatly lower cell proliferation in SNHG1-overexpressing BM-EPCs. However, the modest inhibition was abrogated by co-transfection of miR-181c-5p mimics (Fig. 5A and B). Matrigel assays indicated that the tube length was significantly reduced in the BM-EPCs transfected with the SNHG1 vector, while miR-181c-5p mimics could partly abolish the suppressive effect of SNHG1 on tube formation (Fig. 5C and D). Furthermore, the co-transfection of the miR-181c-5p mimics reversed the inhibitory effects of SNHG1 vector on VEGF expression (Fig. 5E and F). In terms of cell migration, transwell assay indicated that overexpression of SNHG1 decreased the number of migratory cells, whereas overexpression of miR-181c-5p attenuated the SNHG1 vector-induced inhibition of motility of endothelial cells (Fig. 5H and I). These results demonstrated that SNHG1 affected BM-EPCs tube formation via regulation of miR-181c-5p expression.

### LncRNA SNHG1 regulates SFRP1/Wnt3a signaling pathway by interacting with miR-181c-5p

**Fig. 6:**
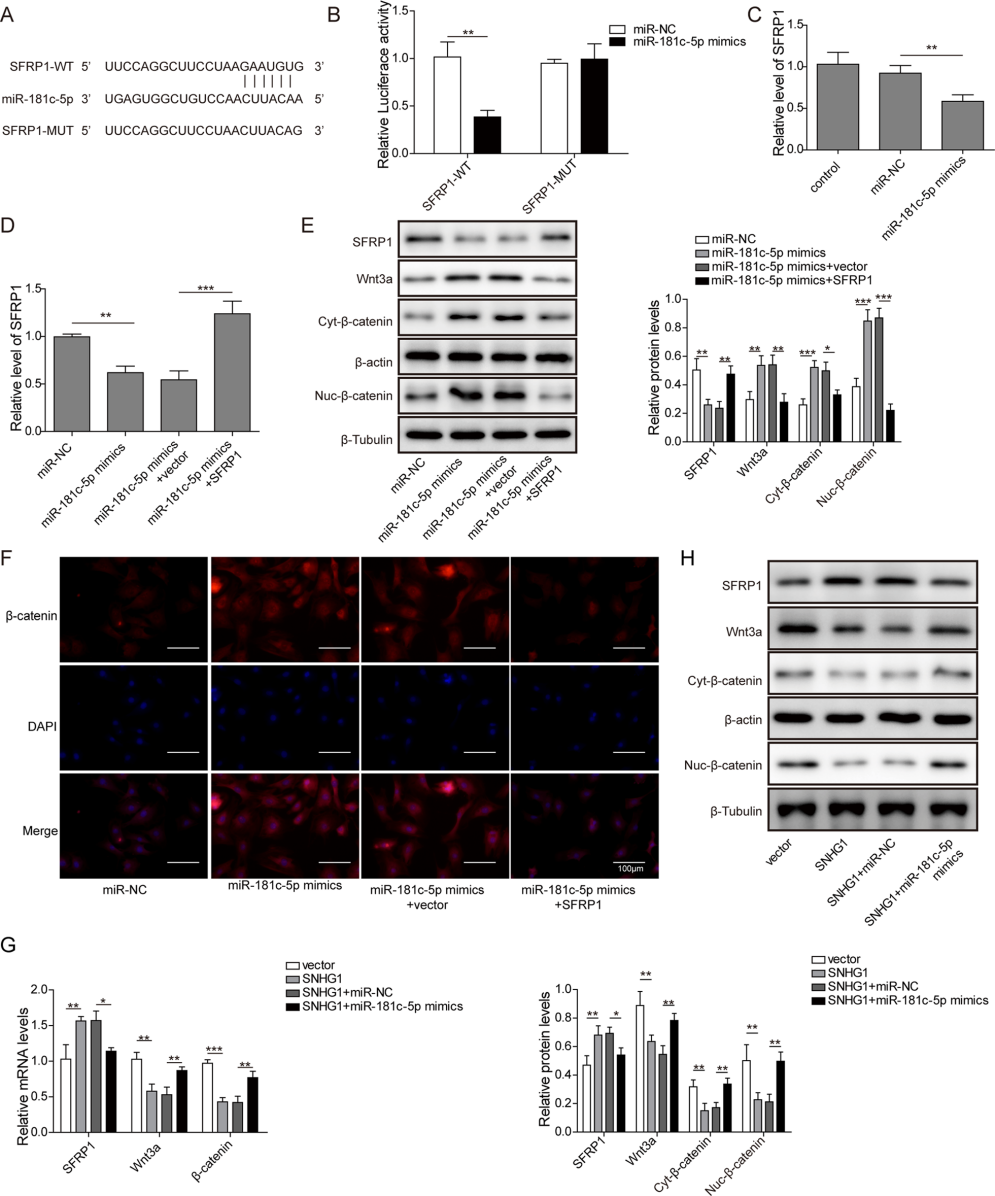
LncRNA SNHG1 regulated SFRP1/Wnt signaling pathway by interacting with miR-181c-5p. **A** StarBase analysis showed the predicted miR-181c-5p binding sites in the 3′ UTR of SFRP1 mRNA. **B** Dual luciferase reporter assay performed to verify the association of miR-181c-5p and SFRP1. **C** The BMSCs were transfected with miR-181c-5p mimics or miR-NC for 48 h, the expression level of SFRP1 was detected by qPCR. **D–F** The BMSCs were transfected with miR-NC, miR-181c-5p mimics, miR-181c-5p mimics with vector NC, miR-181c-5p mimics with SFRP1vector for 48 h. The mRNA level of SFRP1 was detected by qPCR (**D**), the protein levels of SFRP1, Wnt3a and β-catenin were detected with western blot (**E**), the cytoplasmic and nuclear expression of β-catenin were observed by immunofluorescence (**F**). **G** and **H** After transfection with vector-NC, SNHG1 vector, SNHG1 vector with miR-NC, SNHG1 vector with miR-181c-5p mimics for 48 h, the mRNA and protein expression of SFRP1, Wnt3a and β-catenin in BMSCs were assessed through qPCR and western blot, respectively. *P < 0.05, **P < 0.01, ***P < 0.001

Bioinformatics analysis remaindered that the 3′-UTR of SFRP1 may have some complementary sites with miR-181c-5p (Fig. 6A). The results of luciferase reporter assay showed that luciferase activity of SFRP1-WT was reduced after co-transfection of miR-181c-5p mimics as compared to miR-NC (Fig. 6B). However, luciferase activity in SFRP1-MUT groups remained completely unchanged in the presence of miR-181c-5p mimics or miR-NC. In addition, SFRP1 expression was greatly inhibited by miR-181c-5p overexpression (Fig. 6C). To evaluate the interactive impact of miR-181c-5p and SFRP1 on Wnt3a signaling pathway, we transfected MSCs with miR-181c-5p mimics alone or co-transfected with miR-181c-5p mimics and SFRP1 vector. Co-transfection with miR-181c-5p mimics and SFRP1 vector restored SFRP1 mRNA level in BMSCs (Fig. 6D). Meanwhile, miR-181c-5p repressed SFRP1 and Cyt-β-catenin protein expression, but increased Wnt3a and Nuc-β-catenin protein expression, and these effects of were abolished by SFRP1 overexpression (Fig. 6E). Meanwhile, the cytoplasmic and nuclear localization of β-catenin was verified by IF assay, miR-181c-5p overexpression enhanced nuclear localization of β-catenin, which was partial abolished by co-transfection of SFRP1 vector (Fig. 6E). In addition, the BMSCs were also transfected with SNHG1 vector alone or co-transfected with SNHG1 vector and miR-181c-5p mimics, the expression of SFRP1 and Wnt3a were measured by qPCR and western blot. As showed in Fig. 6C–E), SNHG1 overexpression increased SFRP1 expression but down-regulated Wnt3a, cytoplasmic and nuclear expression of β-catenin compared with that of control, whereas co-transfection with miR-181c-5p mimics greatly attenuated these changes. Together, SNHG1 could regulate SFRP1-dependent Wnt3a signaling pathway through sponging miR-181c-5p.

### LncRNA SNHG1 silence alleviates the symptoms of osteoporosis in OVX mice

**Fig. 7:**
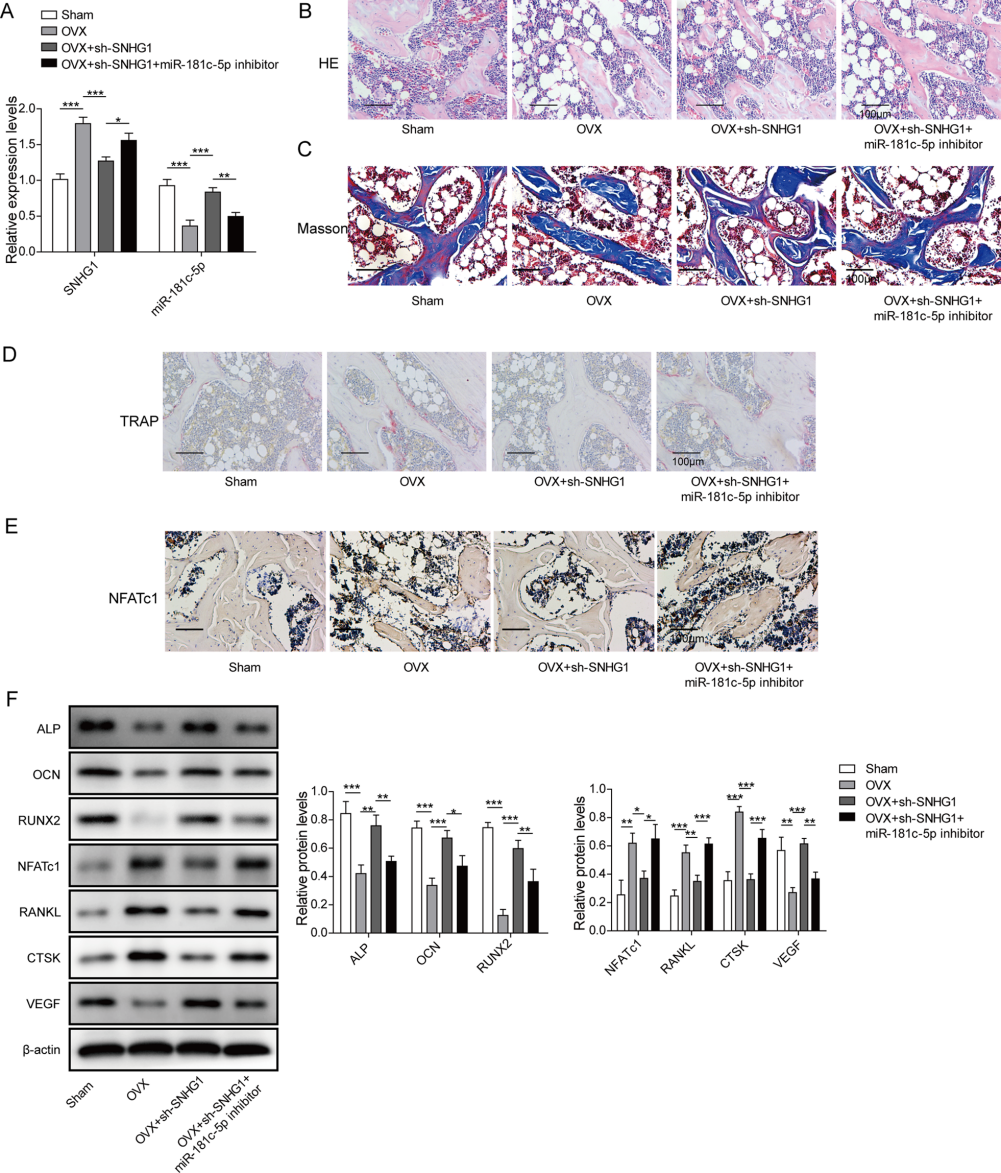
LncRNA SNHG1 silence alleviated the symptoms of osteoporosis in OVX mice. Mice were randomly divided into four groups: Sham, OVX, OVX + sh-SNHG1 and OVX + sh-SNHG1 + miR-181c-5p inhibitor groups. Mice were injected the above and femurs were removed after 8 weeks. **A** The SNHG1 and miR-181c-5p expression levels in femoral tissue were detected through qPCR. **B** Representative HE staining of distal femoral sections showing the bone formation. **C** Representative images of Masson staining in femurs showing the new bone growth. **D** Representative photomicrographs of TRAP staining in distal femora showing the bone destruction. **E** NFATc1 expression was assessed by performing IHC staining. **F** The protein expression of ALP, OCN, RUNX2, VEGF, NFATc1, RANKL and CTSK were measured using western blot. *P < 0.05, **P < 0.01, ***P < 0.001

To validate the roles of the lncRNA SNHG1 in vivo, we established OVX mouse model. After bilateral ovariectomy, mice were injected with sh-SNHG1 or sh-SNHG1 with miR-181c-5p inhibitor. qPCR detection showed that the SNHG1 was downregulated, but the miR-181c-5p was upregulated in femurs of OVX mice injected with sh-SNHG1 compared to these levels in OVX group, however, the increase of miR-181c-5p caused by sh-SNHG1 was diminished after co-transfection with miR-181c-5p inhibitor (Fig. 7A). We then evaluated the new bone formation with histological staining approaches. The HE staining demonstrated that sh-SNHG1 increased the new bone regeneration in OVX mice, but this increase was diminished by miR-181c-5p inhibition (Fig. 7B). Meanwhile, Masson’s staining showed that silencing of SNHG1decreased the collagen deposition at the new bone formation zone and hindered the new bone growth consequently (Fig. 7C). Moreover, TRAP staining displayed that knockdown of SNHG1 prominently reduced TRAP positive cells in OVX mice, while miR-181c-5p inhibitor reversed the effect of sh-SNHG1 (Fig. 7D). Furthermore, IHC staining of the bone tissue samples demonstrated that the upregulation of NFATc1 protein synthesis in OVX mice was significantly suppressed by silencing of SNHG1, however, the inhibitory effect was moderately counteracted by miR-181c-5p knockdown (Fig. 7E). Additionally, it could be observed the markedly increases of ALP, OCN, RUNX2 and VEGF, and significantly decrease of NFATc1, RANKL and CTSK in OVX + sh-SNHG1 group compared with OVX group, while these changes were restored by miR-181c-5p inhibitor transfection (Fig. 7F). These data suggested that SNHG1 knockdown prevented ovariectomy-induced osteoporosis via targeting miR-181c-5p.

### SP1 activates lncRNA SNHG1 transcription

**Fig. 8:**
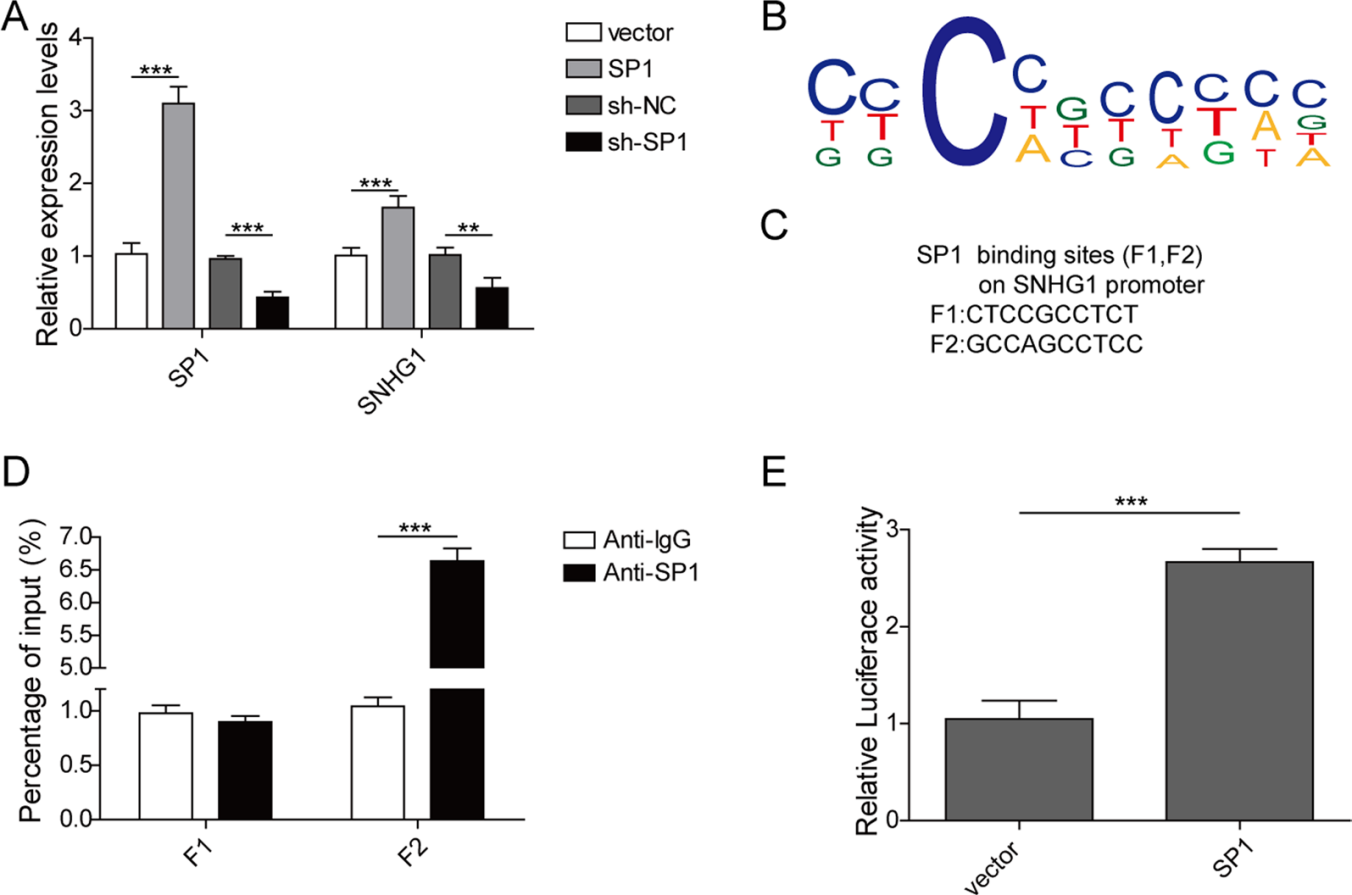
LncRNA SNHG1 was activated by SP1. **A** The BMSCs were transfected with SP1 vector, sh-SP1 or their negative control, the levels of SP1 and lncRNA SNHG1 were measured using qPCR. **B** Predicted positions of putative SP1 binding motif in SNHG1 promoter using JASPAR. **C** Schematic presentation of SP1 binding sites in the promoter region of SNHG1. **D** ChIP-qPCR analysis showed the SP1 occupancy of SNHG1 promoter. **E** Dual luciferase reporter assay was conducted to verify SP1 binding sites on SNHG1 promoter region around F2. **P < 0.01, ***P < 0.001

The qPCR analysis showed that lncRNA SNHG1 expression in BMSCs was up-regulated by SP1 overexpression, but down-regulated by SP1 silencing (Fig. 8A). The JASPAR online database predicted the potential binding sites on the promoter region of lncRNA SNHG1 towards SP1 (Fig. 8B and C). Subsequently, ChIP assay revealed the SP1-binding activity on the lncRNA SNHG1 promoter region around F2 (Fig. 8D). Results of luciferase reporter assays showed that luciferase activity was significantly increased in SP1 vector transfected cells compared with control vector (Fig. 8E). In summary, transcription factor SP1 activated the transcription level of lncRNA SNHG1.

## Discussion

Osteoporosis remains one of the most common bone diseases worldwide (Li et al. 2019). Bone metabolism depends on the regulation between the bone-forming osteoblasts and bone-absorption osteoclasts (Al-Bari and Al 2020; Xu et al. 2018). The osteogenic and osteoclast differentiation of BMSCs serve a major role in bone reconstruction and help maintain a balance between bone formation and bone resorption. Increasing literatures showed that bone vessels play an important role in bone development, remodeling and balance by providing nutrients, growth factors, hormones and chemokines to bone tissue and removing metabolic waste (Hendriks and Ramasamy 2020). As Fu et al. reported, ZEB1 encapsulated liposomes recovered the NOTCH1 activity in skeletal endothelium, leading to angiogenesis-related osteogenesis and reducing bone loss (Fu et al. 2020). In the present study, we focused on the roles and underlying mechanism of lncRNA SNHG1 in bone differentiation and osteoangiogenesis during osteoporosis.

Emerging evidences showed that SNHG1 is correlated with the development and progression of osteoporosis. For example, Xiang et al. reported that SNHG1 level was decreased in a time‑dependent manner during osteogenic differentiation of BMSCs, and its overexpression repressed osteogenic differentiation by sponging miR‑101/DKK1 axis (Xiang et al. 2020). Similarly, Jiang et al. proved that SNHG1 was upregulated in the osteoporosis mice, and suppressed osteogenic differentiation of BMSCs by inhibiting Nedd4-meidated p38 MAPK signal pathway (Jiang et al. 2019). Consistent with previous reports, we found that SNHG1 expression were up-regulated in the serum and femoral tissue of OVX mice. Moreover, overexpression of SNHG1 inhibited the osteogenic osteoclast differentiation of BMSCs and angiogenesis of BM-EPCs, but improved the osteoclast differentiation of BMSCs. It is worth noting that silencing of SNHG1 resulted in the inhibition of migration, proliferation and angiogenesis of vascular endothelial cell (Zhang et al. 2020), further confirming our findings. Importantly, knockdown of SNHG1 in *vivo* promoted bone formation and prevented ovariectomy-induced bone injury, indicating that SNHG1 may be an important factor which cause the aggravation of OP.

SP1 is an important transcription factor in the process of osteogenic/osteoclast differentiation and can promote the expression of different factors in the process of bone differentiation, such as RANKL (Liu et al. 2005), miR-139-5p (Zhang et al. 2017), miR-545-3p (Li et al. 2019), Frizzled-1 (Yu et al. 2016). However, reports on the function of SP1 in osteogenesis/osteoclast differentiation is not completely consistent, which might be related to the transcriptional regulation of genes. Moreover, there have been a few reports suggesting that SP1 is related to the transcriptional activation of SNHG1 (Ding et al. 2020; Zhao et al. 2020). Consistently, by ChIP and dual luciferase report assays, our data have observed that SNHG1 could be activated by SP1 in BMSCs. It is worth to explore whether SP1 can affect the progression of osteoporosis by modulating SNHG1.

LncRNAs could participate in physiological and pathological processes through competitively binding to miRNAs (Yang et al. 2020). Here, we found that SNHG1 could directly bind to miR-181c-5p and down-regulate miR-181c-5p expression. Genetic evidence revealed that miR-181c-5p was a potential biomarker of osteoporosis, it could regulate bone metabolism and differentiation in osteoporosis (Ma et al. 2020). In this study, we confirmed that knockdown of miR-181c-5p suppressed the osteogenic differentiation of BMSCs, whereas overexpression of miR-181c-5p promoted osteoclast differentiation of BMSCs. These findings were also uncovered by Murodumi et al. work (Murodumi et al. 2020). Notably, miR-181c-5p was considered as a anti-angiogenic miRNA which was correlated to impaired angiogenic capacity in diabetes (Morrison et al. 2021). Similar data was found in our research. Overexpression of miR-181c-5p restrained the angiogenesis, migration and proliferation of BM-EPCs, remarkably weakening the promoting effects of SNHG1 overexpression on these phenotypes. From these findings, it could elucidate that miR-181c-5p is a functional target downstream of SNHG1 in osteoporosis.

SFRP1 is one of the most important proteins in the SFRPs family and a negative regulator of human osteoblast and osteocyte survival (Amjadi-Moheb et al. 2018). Our results showed that SFRP1 might be a direct target of miR-181c-5p, and could be positively regulated by SNGH1. This indicated that SNGH1 enhanced the expression of SFRP1 by sponging miR-181c-5p, and thereby regulating the bone remodeling and angiogenesis. Of note, SFRP1 is an antagonist of the Wnt/β-catenin signaling pathway; it can inhibit the downward transduction of Wnt and transduction protein (Holdsworth et al. 2018). The Wnt/β-catenin pathway promotes bone mineralization stimulating proliferation, differentiation, and survival of osteoblasts; it also inhibits osteoclast differentiation and osteocyte activity (Duan and Bonewald 2016; Vazquez-Villegas et al. 2021). Studies have reported that Wnt/β-catenin signaling pathway serves a vital role in OP and that the inhibition of this pathway can improve bone density in an ovariectomized (OVX) rat model (Liu et al. 2019; Tan et al. 2015). Wnt/β-catenin pathway also plays important roles in angiogenesis and vasculogenesis (Shen et al. 2021). In this study, we found that overexpression of miR-181c-5p could activate Wnt/β-catenin signaling by negatively regulating SFRP1 expression. Importantly, it was also demonstrated that suppression of the Wnt/β-catenin signaling pathway as a result of SNHG1 overexpression was partially reversed by miR-181c-5p mimics. These results suggested that SNHG1/ miR-181c-5p axis may regulate bone remodeling and angiogenesis by targeting SFRP1, which is an antagonist of the Wnt signaling pathway.

## Conclusions

In summary, our findings identified that lncRNA SNHG1 regulated by SP1 play a promoting role in osteoporosis development that suppressing osteogenic differentiation and angiogenesis but facilitating osteoclast differentiation by modulating SFRP1-mediated Wnt signaling through sponging miR-181c-5p. These results revealed a novel mechanism of SNHG1 in the pathogenesis of osteoporosis, meanwhile, it also suggested that inhibition of SNHG1 might be a therapeutic strategy for osteoporosis treatment.

## Data Availability

The datasets used or analyzed during the current study are available from the corresponding author on reasonable request.

## Abbreviations

ALP: Alkaline phosphatase
ARS: Alizarin red S
OVX: Ovariectomy
BMSCs: Bone marrow mesenchymal stem cells
EPCs: Endothelial progenitor cells
LncRNA: Long non-coding RNAs
MiRNAs: MicroRNAs
SFRP1: Frizzled-related protein-1
EGF: Epidermal growth factor
FGF: Fibroblast growth factor
VEGF: Vascular endothelial growth factor
FBS: Fetal bovine serum
M-CSF: Macrophage-colony stimulating factor
RANKL: Receptor activator of nuclear factor (NF)-κB (RANK) ligand
shRNA: Short hairpin RNA
PBS: Phosphate-buffered saline
PFA: Paraformaldehyde
RIP: RNA binding protein immunoprecipitation
ChIP: Chromatin immunoprecipitation
HE: Hematoxylin and eosin
OCN: Osteocalcin
RUNX2: Runt domain transcription factor 2
TRAP: Tartrateresistant acid phosphatase
CTSK: Cathepsin K
NFATc1: Nuclear factor of activated T cells cytoplasmic 1
